# Generalized Circuit Topology of Folded Linear Chains

**DOI:** 10.1016/j.isci.2020.101492

**Published:** 2020-08-22

**Authors:** Anatoly Golovnev, Alireza Mashaghi

**Affiliations:** 1 Medical Systems Biophysics and Bioengineering, Leiden Academic Centre for Drug Research, Faculty of Science, Leiden University, 2333CC Leiden, the Netherlands

**Keywords:** Polymer Chemistry, Theoretical Chemistry, Molecular Network, Biophysics

## Abstract

A wide range of physical systems can be formally mapped to a linear chain of sorted objects. Upon introduction of intrachain interactions, such a chain can “fold” to elaborate topological structures, analogous to folded linear polymer systems. Two distinct chain-topology theories, knot theory and circuit topology, have separately provided insight into the structure, dynamics, and evolution of folded linear polymers such as proteins and genomic DNA. Knot theory, however, ignores intrachain interactions (contacts), whereas chain crossings are ignored in circuit topology. Thus, there is a need for a universal approach that can provide topological description of any folded linear chain. Here, we generalize circuit topology in order to grasp particularities typically addressed by knot theory. We develop a generic approach that is simple, mathematically rigorous, and practically useful for structural classification, analysis of structural dynamics, and engineering applications.

## Highlights

• We develop a topology framework for folded linear chains
• Our theory accounts for both intra-chain contacts and chain entanglement
• The topology framework provides a 3D description of a chain spatial conformation
• A quantitative measure of topological similarity of different chains is developed

## Graphical Abstract

**Figure:**
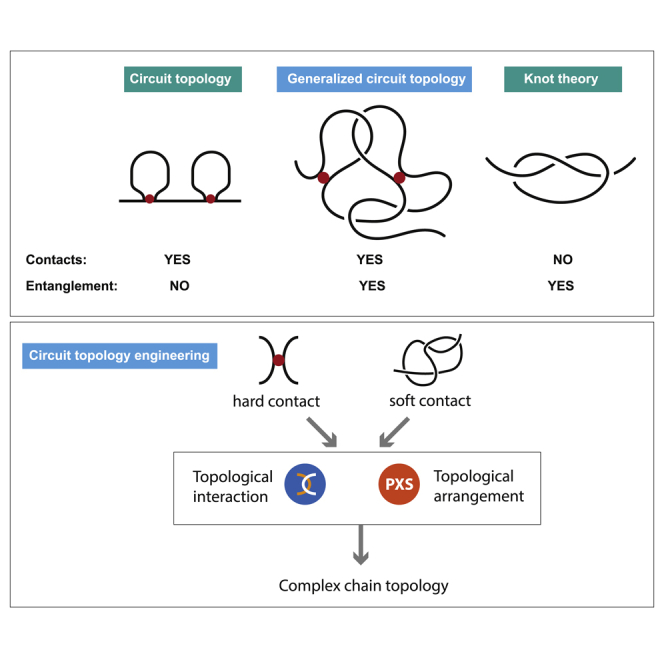

## Introduction

In order to describe the immense structural diversity of proteins, nucleic acids or other linear molecular chains, the concept of circuit topology was recently introduced to formally categorize the arrangement of intrachain contacts (Heidari et al., 2020) (Scalvini et al., 2020) (Mashaghi et al., 2014). It analyses pairwise relationships of intrachain contacts and shows that only a limited number of arrangements of two contacts are topologically distinct. For binary contacts, those arrangements are parallel (P), series (S), and cross (X). The approach allows for topology classification and for extracting topological information from a folded chain, which is suggested to be useful in understanding folding pathways and kinetics as well as in predicting functional and evolutionary relatedness of biomolecules (Heidari et al., 2017) (Heidari et al., 2019) (Mugler et al., 2014) (Nikoofard and Mashaghi, 2016) (Nikoofard and Mashaghi, 2018) (Satarifard et al., 2017) (Schullian et al., 2020). The approach is, however, limited as it does not include chain entanglement, a topic that is addressed by knot theory (Adams, 2004). Application of knot theory to physics and biology has led to important insights into various physical systems ranging from polymers to fluid dynamics and optics (Dennis et al., 2010) (Flapan, 2000) (Kleckner et al., 2016) (Larocque et al., 2018) (Leach et al., 2004) (Moffatt, 1990) (Sukowska et al., 2008) (Sukowska et al., 2012) (Sukowska et al., 2018) (Wang and Zhang, 2018) (Zhang et al., 2019) (Ziegler et al., 2016). However, current knot theory ignores intrachain contacts and, consequently, cannot inform on contact arrangement (circuit topology), limiting its application to topological analysis of linear chains with intrachain contacts, which are typical for biomolecules. Furthermore, introduction of contacts allows for establishing chain crossings that are not invariant in the knot representation yet are important topological features when physics of the chains are studied.

In the present paper, we extend the idea of circuit topology to include entanglement of chain segments. We propose a new notation that codes the conformation of a folded linear chain, extend the definition of “contacts” to include chain crossing, and then use circuit topology to analyze the arrangement of contacts. The new notation is a string of letters, where each letter is related to an intrachain contact. It allows one to employ the well-developed and ideologically transparent framework of combinatorics to description of a molecular chain 3D structure. Generalized circuit topology is simple but generic and uniquely provides a general topological classification and analysis framework for any folded chain including a wide range of linear biomolecules; to classify them based on their spatial structure so that all the chains within one class possess common features, even though they have different chemical structure. It also provides experimentally measurable quantities, which allows one to map chain folding processes onto the topological space and to correlate topological features with physicochemical properties of molecules.

Circuit topology describes an abstract linear chain that is not necessarily a biopolymer. The linear chain could be a spin system or a cascade of chemical reactions (Ghafari and Mashaghi, 2017), or it might even be not a physical entity but a logical chain. One example is cascade correlations and patterns in machine deep learning (Fahlman and Lebiere, 1990; Veit et al., 2016), where circuit topology contacts correspond to logical connections of elements (units). However, in the present paper we mainly refer to biomolecules as examples for two reasons. First of all, it is an obvious and very broad application, which is important to discuss. Second, we want to visualize the new development of the theory to avoid misunderstanding. If any formal transformation is not intuitively clear, one can simply take a rope and tie it as depicted in the corresponding figure.

In the following sections, we briefly overview the basics of circuit topology and introduce the new notation and use this notation to describe a given chain entanglement. Next, we summarize our findings obtained for a single chain. We then discuss measures of comparison of different chains and analyze our results in context of knot theory and possible experimental measurements.

## Results

### String Notation

**Figure 1:**
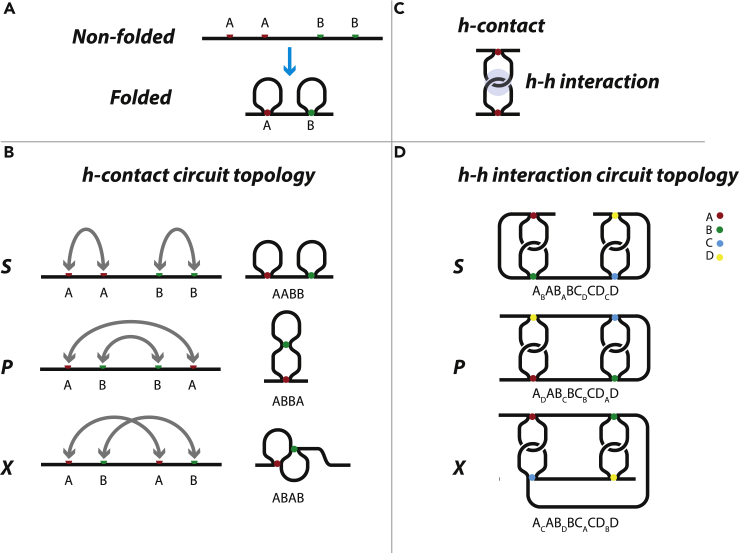
Hard Contacts and Their Interactions Left panels (A and B) show the formation of h-contacts and their possible arrangements, namely, in series (S), in parallel (P), in cross (X). All contacts are pairwise, i.e., connect two chain segments. Right panels (C and D) show an interaction of h-contacts and sketch all possible arrangements of pairs of interacting h-contacts.

Let us define an h-contact, or a hard contact, as an intrachain contact where different parts of a chain are held together via gluing two contact sites, Figure 1A. The origin of the holding force is irrelevant (e.g., it could be a chemical bond). The contact site cannot move along the chain. To break an h-contact, one has to break the corresponding bond. Each h-contact gives rise to an h-loop. Figure 1B contains three chains. Each chain has two h-contacts, **A** and **B**, which are marked with colored balls. Each contact has two sites: “left” site and “right” site. Let us consider the upper chain. If we start at one of its ends, say from the left end, and move along the chain, we see: left tail, then contact **A** (red ball), then we move along the loop and approach another site of contact **A**, then contact **B** (green ball) from the left, the loop, then contact **B** from the right, then the chain ends. If we leave only contacts, we have **AABB**. This combination is called S-structure and describes two h-contacts, or two h-loops, placed in series (hence the name) (Mashaghi et al., 2014). H-contacts are written in bold in order to distinguish them from another type of contacts introduced below in section “S-Contacts”.

Let us do the same procedure for the next chain in Figure 1B. What do we see as we move along the chain? Chain tail, contact **A**, half-loop, contact **B**, loop, contact **B**, half-loop, contact **A**, another chain tail. Leaving only contacts we have **ABBA**. This is P-structure because the loops are in parallel. Similarly, the last chain at the bottom of Figure 1B gives **ABAB**, which is X-structure or cross structure. Note, **AABB**, **ABBA**, **ABAB** comprise all the different combinations possible for two non-ordered pairs of letters (**ABAB** and **BABA** are equivalent because each contact can be given any name). It proves that only these three structures exist for a pair of two-point contacts, thus the “circuit topology” classification is complete. This statement can be visualized. Consider a chain with one h-loop, i.e., with one h-contact. It has three parts: the left tail, the loop, and the right tail. We want to form another loop. We take, say, the right tail. Where can it go to form a contact? It has three options: either to the left tail (P-structure), or to the loop (X-structure), or to itself (S-structure). The same procedure with the loop gives P- and X-structures.

Unlike S- and X-structures, P-structure gives rise to a kind of hierarchy of contacts in the sense that one contact is internal to another; **ABBA** means that contact **B** lies inside contact **A**. It can be additionally specified (Mashaghi et al., 2014) as P^–1^-structure.

Circuit topology can be extended to three-point contacts, which though is rarely needed in practice, especially while dealing with biomolecules (Mashaghi et al., 2014) (Schullian et al., 2020). Three-point contacts are considered as an extreme case when sites of two-point contacts are merging together, and hence their sequence is not clear, **A(AB)B** versus **A(BA)B**, and **(AB)BA** versus **(BA)BA**. In this case, one should decide against the X-structure for consistency purposes and also because P- and S-structures are easier to handle, as will be seen in the next chapters. The corresponding contacts are called concerted contacts and denoted as CS, concerted series, and CP, concerted parallel.

An h-h interaction occurs when h-loops hook to each other, whereby posing restrictions on chain motion as shown in Figure 1C. Such interaction is ideologically different from an h-contact and shown as a subscript that falls within the corresponding contact. Figure 1D shows all possible pairwise interactions of four h-contacts. Note that the circuit topology rules can be applied not only to contacts but also to chain segments. The diagram of h-h interaction in series can be split into two segments, **A**_*B*_**AB**_*A*_**B** and **C**_*D*_**CD**_*C*_**D**, which are in series. It provides a bottle neck where the chain can be cut in parts with just one cut. Similarly, the diagram in parallel consists of **A**_*D*_**AD**_*A*_**D** and **B**_*C*_**BC**_*B*_**C** in parallel, which can be separated with two cuts that remove the middle segment. Note that in these examples each letter belongs only to one segment. And the last diagram in cross cannot be split into independent segments and hence cannot be separated with just two cuts. It is easy to see for a small number of contacts but gets harder to visualize when the contact number rises and so the circuit topology formalism is needed.

**Figure 2:**
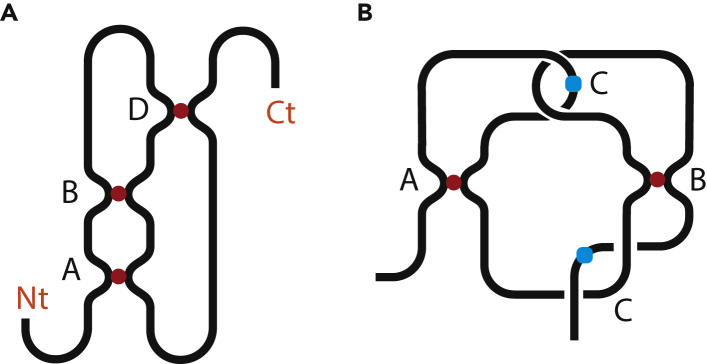
Illustration of Several Contacts in a Chain (A) An example of a chain with h-contacts, **NtABDBADCt**. The chain ends are distinguishable. (B) A chain with both h- and s-contacts, ${A\text{C}}_{+B}^{+e}$**AB**_+*A*_**B**C. Contact sites are color-coded.

Listing contacts and subscripts in the sequence they appear on the chain gives rise to a string. This notation does not specify from which end the chain is read. Hence in principal, the string can be read both from left to right and from right to left. If there is a chemical difference between the chain ends, it can be specified on the string. In case of proteins, it is **Nt**, i.e., N-terminus, and **Ct**, i.e., C-terminus; e.g., **NtABDBADCt**. This string describes the chain from Figure 2A. It has three h-contacts, **A**, **B**, and **D**. A relative position of each contact pair can be easily seen by considering only the pair in question, ignoring other contacts. Indeed, two contacts can be connected by a simple chain segment with no structure or by a strongly entangled and knotted segment. But it does not affect the relative position of the contacts in question. This is why circuit topology considers pairwise relationships. For example, for contacts **A** and **D**, **Nt***A***B***D***B***AD***Ct** → **ADAD**, which is X-structure. In other words, finding out a relative position of contacts is equally simple for short chains with few contacts and long chains with many contacts.

One of the advantages of this notation is that all changes in the contact structure of the chain can be dealt with a help of combinatorial analysis by simple rearrangements of the string letters. For example, if there are *n* contacts, it is a simple combinatorial task to find the number of all possible configurations, $\left(2n\right)!/\left(2^{n}n!\right)$. Indeed, *n* contacts give rise to 2*n* contact sites, which defines the string length. Hence, the question is how many different ways are there to distribute *n* pairs of letters over 2*n* sites. The first pair has all 2*n* sites available and can be positioned in $\left(\begin{array}{c} 2n\\ 2 \end{array}\right)\equiv \frac{\left(2n\right)!}{\left(2n-2\right)!2}$ ways. The next pair has 2n−2 contact sites available, which gives $\left(\begin{array}{c} 2n-2\\ 2 \end{array}\right)\equiv \frac{\left(2n-2\right)!}{\left(2n-4\right)!2}$ ways. And so on for all *n* pairs. Because it is irrelevant in which sequence the letters are placed on the string, the result should be divided by n!:

$$\frac{1}{n!}\left(\begin{array}{c} 2n\\ 2 \end{array}\right)\left(\begin{array}{c} 2n-2\\ 2 \end{array}\right)\left(\begin{array}{c} 2n-4\\ 2 \end{array}\right)\cdots =\frac{1}{n!}\frac{\left(2n\right)!}{\left(2n-2\right)!2}\frac{\left(2n-2\right)!}{\left(2n-4\right)!2}\frac{\left(2n-4\right)!}{\left(2n-6\right)!2}\cdots =\frac{\left(2n\right)!}{n!2^{n}}\text{.}$$

If an h-contact breaks, the corresponding letter just disappears from the string. If two chains merge, their strings merge in the same manner. These and other changes in a chain structure were recently considered in Schullian et al. (2020) by using a connectivity matrix *S* that shows which contact sites are connected. Here we will not consider it again and duplicate the calculations but will demonstrate the equivalence of the notations. This allows one to model molecular engineering operations commonly seen in biology or used in laboratories for synthesizing desired folded molecules.

Rows and columns of a connectivity matrix *S* are ordered in exactly the same manner as a string is ordered. Thus, the row number *k*, the column number *k*, and the string's letter number *k* all correspond to the contacts site number *k*. Hence, $S_{nl}=1$ says that contact sites number *n* and *l* are connected. It implies that the string's letters number *n* and *l* should coincide. If $S_{nl}=0$, contact sites number *n* and *l* are not connected.

**From string to matrix.**

Let us consider **ABAB** which has 4 contact sites. Contact **A** says that site 1 and site 3 are connected, so $S_{13}\equiv S_{31}=1$. Similarly, contact **B** says that $S_{24}\equiv S_{42}=1$. Other elements of *S* are 0. Hence,

$$S=\left(\begin{array}{cccc} 0 & 0 & 1 & 0\\ 0 & 0 & 0 & 1\\ 1 & 0 & 0 & 0\\ 0 & 1 & 0 & 0 \end{array}\right)$$

**From matrix to string.**

Each contact is assigned a name, i.e., a letter. Each column has only one letter. Each column is replaced by the letter it contains. For example,

$$S=\left(\begin{array}{cccc} 0 & 0 & 0 & 1\\ 0 & 0 & 1 & 0\\ 0 & 1 & 0 & 0\\ 1 & 0 & 0 & 0 \end{array}\right)\to \left(\begin{array}{cccc} 0 & 0 & 0 & A\\ 0 & 0 & 1 & 0\\ 0 & 1 & 0 & 0\\ A & 0 & 0 & 0 \end{array}\right)\to \left(\begin{array}{cccc} 0 & 0 & 0 & A\\ 0 & 0 & B & 0\\ 0 & B & 0 & 0\\ A & 0 & 0 & 0 \end{array}\right)\to ABBA$$

### S-Contacts

**Figure 3:**
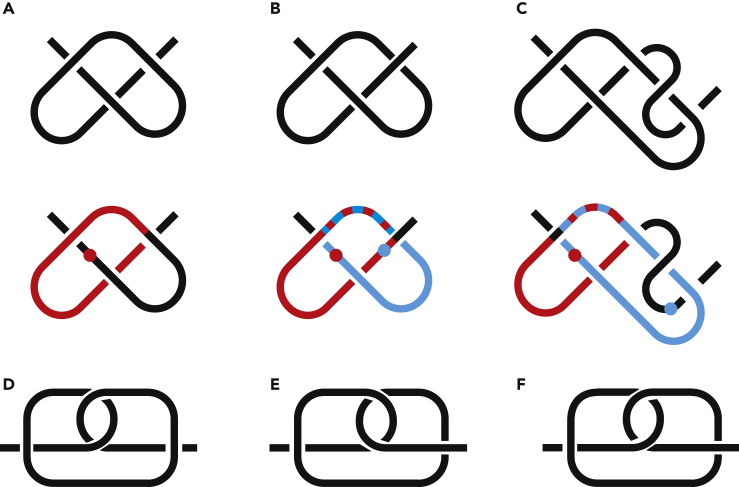
Chain Entanglement and Soft Contacts (A–F) (A) and (D) each are disentangled and hence untie if the chain ends are pulled apart; (B) and (E) are A^+*e*^A; (C) and (F) are A^+*o*^A. Here, (A)–(C) are 3D structures, whereas (D)–(F) are their flat symmetric representations. The approximate position of a chain segment enveloped by another segment is shown in color.

In this chapter, we do not consider h-contacts but will focus on chain entanglement and will incorporate it into the circuit topology framework. Intrachain contacts always restrict free motion of the chain because they lock the mutual positions of different segments. We adopt the idea that the opposite also holds, i.e., a restriction of motion should generate a contact, even if there are no h-contacts. In order to lock one segment in position, the chain should go around this segment, enveloping it by forming a loop around it. Then, the loop should get fixed, so it does not untie spontaneously. Figure 3A shows a loop around a chain segment. The loop is not stable; it will untie. To fix it, a chain tail should go through it. It can be done in two ways shown in Figures 3B and 3C. Note that this chain tail also gets enveloped by another chain segment. So, there are two chain segments enveloped by two loops. These segments define the contact sites of an s-contact, or a soft contact. Because loops are not flat, these sites cannot be precisely located in space, but what matters is their relative position along the chain, i.e., the position in the string, which is well defined. Two sites on an h-contact are close to each other in space (in case of real chains, not logical chains). Two sites of an s-contact can be far apart from each other. S-contacts are fictious and designed to describe a chain entanglement. In contrast to h-contacts, s-contacts are not marked in bold, e.g., AA, ABAB.

To distinguish between Figures 3B and 3C, one has to introduce chirality. This property is universal and does not depend on the chain orientation, i.e., which end of the chain we go from, left to right or right to left. However, once the chain orientation is chosen, it should be the same for all loops. The positive direction of a loop is defined by the right-hand rule, namely, the four fingers curl as the loop goes based on the chain orientation, so the right thumb points in the positive direction. If the chain shifts in the positive direction while passing the loop, then the loop is positive, otherwise negative. The left loop in in Figure 3D is positive, while the right loop in Figure 3D is negative.

In Figures 3A–3C, we go from the left tail, then over the loop shown in red, and then to the right tail. There are only three possibilities what the right tail can do. It (1) does not go through the loop, (2) goes through the loop in the positive direction, (3) goes through the loop in the negative direction. If we go from the right tail, we should obtain the same result. However, the chirality of the other loop in Figure 3C is not clear because the chain is twisted. In general, in 3D, the definition of a loop might be ambiguous because chain segments are not close to each other. Each contact in Figures 3B and 3C has two loops and hence can be homotopically deformed into two loops in series hooked to each other. A homotopic deformation means that the chain ends are far away, so the structure and content of the loops do not change during deformation. The structures in Figures 3D–3F should be seen as flat. The positive direction of all loops is toward the reader if we go from left to right. If the loops have different chirality, as in Figure 3D, it is disentangled. This can be seen by pulling the lowest part of the chain in the middle up. It cannot be done in Figures 3E and 3F owing to the crossing of the right loop. To form an s-contact, the loops must be the same chirality, either both negative or both positive. All the loops in Figures 3E and 3F are positive. The difference between them lies in the direction in which segments pass through loops, i.e., how loops hook to each other. In Figure 3E this direction coincides with the loop chirality (pointed toward the reader if we go from left to right) and therefore the contact is called even. In Figure 3F, it is the opposite direction, so the contact is called odd. Figures 3B and 3E are homotopic, i.e., it is the same system of loops. Similarly, Figures 3C and 3F are also equivalent.

Figure 3B reads A^+*e*^A, where “e” stands for “even” and defines the s-contact symmetry, whereas “+” shows its chirality. The superscript is put only once at the first appearance of the contact. It keeps the notation simple, and also, we want to keep the information about the contact inside the notation for the contact. It will be helpful in Section “Quantification of a String”. Figure 3C reads A^+*o*^A, where “o” stands for “odd.” These are all simple cases involving one circle around a chain segment. In principle, there could be more complicated possibilities involving several circles around a chain segment in Figures 3A–3C, which corresponds to twisting loops in Figures 3D–3F. Such circling or twisting forms new loops; therefore, it is reasonable to expect that it creates new s-contacts. Let us consider it.

**Figure 4:**
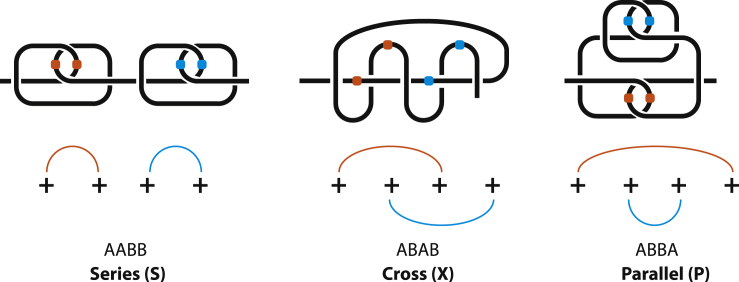
All Possible Pairwise Even Entanglements of Four Positive Loops, A^+*e*^AB^+*e*^B, A^+*e*^B^+*e*^AB, A^+*e*^B^+*e*^BA Contact sites are marked with colored balls. For a similar X-structure see also Figure 10.

**Figure 5:**
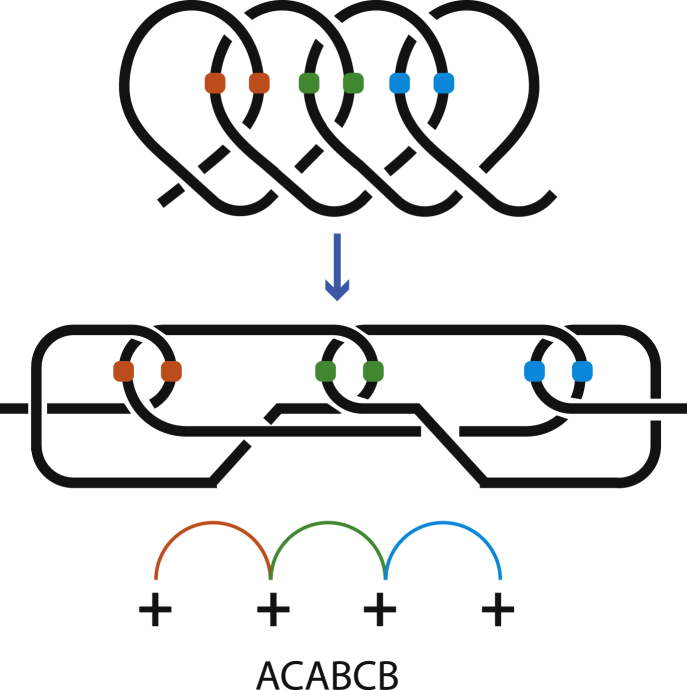
Four Positive Loops are Hooked Even The upper panel is a visual representation by an entangled rope. Below is a schematic. Two left-most loops comprise contact A (red), which hooks contact B (blue) consisting of two right-most loops. This entanglement creates contact C (green).

One can form a loop by twisting a chain. An h-contact forms an h-loop. An s-contact consists of two loops that are collectively called an s-loop. Any messy entangled chain can be split into a set of hooked loops (and h-loops, if any). As mentioned above, circuit topology focuses on pairwise relations of contacts, because a relation of two contacts is independent from other contacts. Let us consider a conformation with four positive loops in Figure 4 and entangle them to form 2 even s-contacts. If we entangle the first two loops and the second two loops (1-2 3-4), we get AABB, so 2 s-contacts are in series. Here we do not write the superscripts because currently the information they contain is not relevant. The resulting AABB structure is steady, i.e., the s-contacts will not disentangle or change qualitatively if the chain ends are pulled apart. They are invariant. The two other options of entanglement are (1-4 2-3), which gives ABBA, and (1-3 2-4), which gives ABAB, which is similar to Figure 3B but with two circles around the chain (i.e., A^+*e*^B^+*e*^AB). We see that s-contacts can be only in series, or in parallel, or in cross. Any other possible entanglement of loops leads to one of the same three configurations: S, P, X. It can be seen in practice by tying a rope or theoretically by looking at Figure 5, which shows four positive loops hooked even (i.e., not odd) in a row. The crossings in each pair of loops are the same as depicted in Figure 3E. To account for different symmetry/chirality of the loops and resulting s-contacts means to consider different crossings. All possible crossings are presented in Figures 3D–3F. Therefore, any entanglement can be represented as a set of structures from Figure 3 and their negative counterparts connected in series, in parallel, or in cross. This is a consequence of the fact that pairwise relations are sufficient. Because loops can disentangle if their crossings correspond to Figure 3D, loops are not invariants. But s-loops are invariants. Therefore, it is more convenient to describe a chain entanglement in terms of s-contacts, not loops.

To derive a string, we go along the chain and write down the “events” we account. A contact is an event. A chain segment passing through an h- or s-loop is also an event that is marked as a subscript. For example, let us take the right tail in Figure 3C and pull it backward to the left, into the loop next to the left tail. This procedure forms a slip-knot. The string from left to right is A^+*o*^A_+*A*_, or from right to left is _+*A*_A^+*o*^A. The superscript +*o* means that the tail exits the contact in the negative direction, whereas the sign in the subscript says that the segment passes through the contact in the positive direction. Therefore, if we pull the chain ends, the chain should disentangle, which is what is expected from a slip-knot. Such subscripts are the only way that s-contacts can disappear. Speculatively, there could happen that A^+*o*^A_+*A*_ unties (i.e., disentangles) spontaneously. Such a transition changes the topology of the system. The transition can be hindered by, for example, forming an h-contact on the loop of the slip-knot. The string will become A^+*o*^A**BB**_+*A*_, where contact *B* makes it harder to pass through the s-contact and disentangle the chain. To quantify how much harder it will be, one has to account for various non-topological properties such as the loop geometrical size and the chain persistence length in the case of polymeric chains. This is beyond the scope of the present paper and will be considered in future studies. Here, we would like to acknowledge the possibility of addressing such questions in the framework of our approach. In contrast, knot theory cannot deal with slip-knots because they are removable by Reidemeister moves.

The difference between s-contacts and subscripts is that, in s-contacts, two loops are locked together. A subscript is one loop that appears only when there is already a contact and a new s-contact cannot be formed. A subscript is kind of a half of an s-contact. Usually, subscripts describe entanglement involving h-contacts. Entanglement of s-contacts involves no subscripts, see, for example, Figure 5, where two s-contact, i.e., four loops, are entangled, which gives rise to another s-contact named C. For comparison, two similarly entangled h-contacts read **A**_+*B*_**AB**_+*A*_**B**.

Owing to s-contacts chirality, A^+*e*^B^–*e*^BA and A^+*e*^B^+*e*^BA are two different, although similar looking, structures. The same chirality of two s-contacts in parallel means that the chain is more braided. _–*A*_A^+*e*^B^+*e*^BA_–*A*_ is the most typical shoelace knot where the shoe is put into s-loop B. The segment _–*A*_A^+*e*^⋯ means that pulling by the chain end we untie the contact. _–*A*_A^+*e*^B^–*e*^BA_–*A*_ is a less efficient version of the shoe lace knot because it is less braided; hence, it is less tough. Also, A^+*e*^A_–*A*_ and, of course, its symmetric A^–*e*^A_+*A*_ are widely known as a slip knot, A^+*e*^A is a thumb knot, A^+*o*^A is a figure eight knot.

Let us consider Figure 2B, which reads ${A\text{C}}_{+B}^{+e}$**AB**_+*A*_**B**C. The approximate position of the sites of contact C is shown with a blue ball. The segment passing through contact **A** is circled around but not fixed, which is similar to Figure 3A. Therefore, it does not give rise to an s-contact. Contact C and subscript _+*B*_ appear simultaneously. One can write it either ⋯C_*+B*_⋯Aaden or ⋯_+*B*_C⋯Aaden. The rule is to put it inside the corresponding contact, so ⋯C_+*B*_⋯Aaden is correct. If contact **A** breaks, it just disappears form the string, ${A\text{C}}_{+B}^{+e}$**AB**_+*A*_**B**C→${\text{C}}_{+B}^{+e}$**BB**C. If then contact **B** breaks, ${\text{C}}_{+B}^{+e}$**BB**C→C^+*e*^C, which is Figure 3B. If we read the chain from another end, the string should be C^+*e*^**B**_+*A*_**BA**_+*B*_C**A**. We write this string based on the illustration in Figure 2B. However, one can go the other way around. The string along contains all the information necessary to draw Figure 2B.

### Summary of the Rules

An h-contact is a connection of different parts of the chain due to a non-topological reason, for example, by a chemical bond. S-contacts appear because of a topological reason and are designed to describe an entanglement of the chain. Mutual connection of different contacts and locking of free ends are captured by subscripts.

A string is a sequence of events, namely, h-contacts, s-contacts, and chain segments passing through a contact, which are listed in the order as they appear along the chain as subscripts. Contacts are named with letters. Each letter appears exactly two times on the string (disregarding subscripts). In reverse, any string consisting of pairs of letters corresponds to a chain. S-contact can be odd and even.

**Figure 6:**
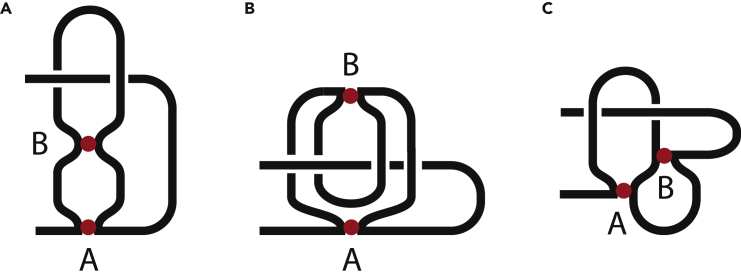
Examples of Interactions between Hard and Soft Contacts **ABBA**_–*B*–*A*_ (A) and **ABBA**_+*B*_ (B). If contact **B** breaks, the letter **B** disappears from the string and the resulting structures are different. **ABAB**_+*A*_ (C). If contacts **A** and **B** break, the string disappears, i.e., the chain disentangles.

The h-h and h-s contact interactions give rise to subscripts. We note that s-s contact interaction generates new s-contacts. If the chain passes through several contacts in a row, all these events have to be marked as the subscript. If two h-contacts are in parallel, the chain can pass through them simultaneously; see Figures 6A and 6B. It is not the case for h-contacts in series, Figure 2B, and h-contacts in cross, Figure 6C.

**Figure 7:**
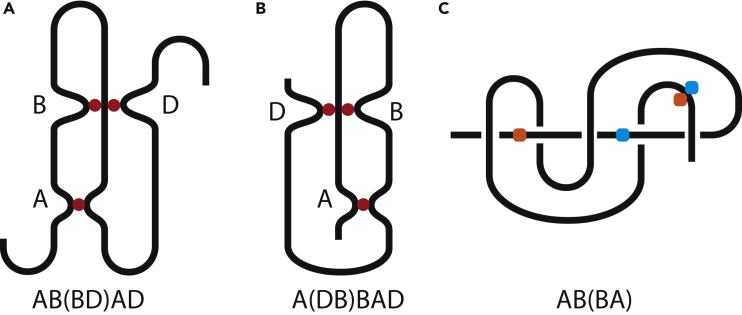
Examples of Concerted Contacts (A and B) Concerted h-contacts in the chains resembling Figure 2A. (C) Concerted s-contacts. The chain resembles Figure 4 middle panel.

If two h-contacts share a contact site (or if two h-contacts are too close to each other and thus not resolvable in experiments), they give rise to concerted contacts. Let us consider the chain from Figure 2A and “merge” contacts **B** and **D**. Two arrangements are possible as shown in Figures 7A and 7B. The parentheses in the string indicate that the sites of different contacts touch the current chain segment simultaneously. Note that we avoid cross configurations of concerted contacts, e.g., **AB**(**DB**)**AD** is wrong.

Concerted s-contacts arise when the chain passes through the loop shared among two s-contacts. Figure 7C shows an example. The right tail passes through the loop and thereby fixes the two s-contacts simultaneously. Compare it with Figure 4 middle panel. Also, the reader is encouraged to tie and compare A^*e*^B^*e*^(BA) and A^*o*^A, which are different by one twist of the chain. Here we do not specify their chirality because it is not important for the purpose of this example.

The circuit topology rules can be applied not only to contacts but also to chain segments. Splitting the chain into such segments helps visualize the overall structure of the chain, to see which segments are hidden inside a blob and which segments are accessible from the outside. For example, ABABCDCD consists of two blobs in series, ABAB and CDCD.

Generalized circuit topology provides a convenient method of counting and analyzing possible 3D configurations. The string notation can be viewed as a “translation” of a chain conformation into the language of combinatorics. We demonstrated it by the example of counting the total number of all possible configurations of a chain with *n* h-contacts, which is $\left(2n\right)!/\left(n!2^{n}\right)$. The same counting is in principle applicable to s-contacts. Also, one can easily see the chain topological symmetry. One example, which will be useful in section “Quantification of a String”, is if a string has a mirror-symmetry, i.e., **ABCDE EDCBA**, it means that all contacts are in parallel. Indeed, any pair of letters is like **ABBA**. If the symmetry is like **ABCDE ABCDE**, then all contacts are in cross. And if a string can be split into pairs of letters like **AA BB CC DD EE**, then all contacts are in series. A more detailed analysis is, although interesting, beyond the scope of the present paper.

**Figure 8:**
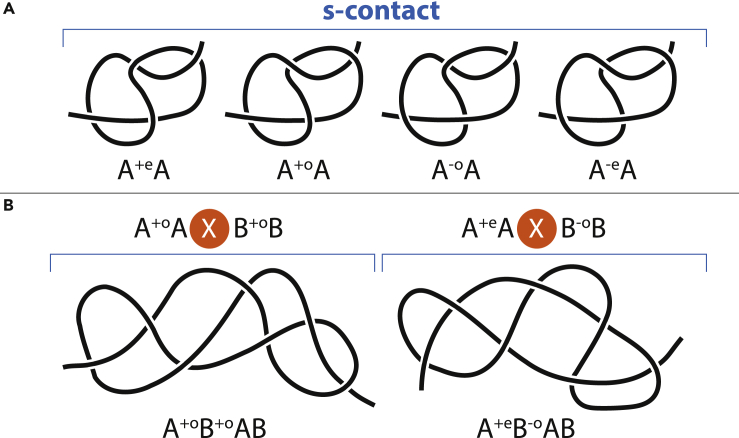
Soft Contacts (A and B) (A) provides a (less schematic) representation of all possible s-contacts. (B) gives examples of more complicated entanglement described in terms of circuit topology. The two chains are generated when two s-contacts are combined in cross, which is X configuration. Examples for P and S configurations of two s-contacts are given in Figure 9. The left configuration in Figure 9 is a series combination of A^+*e*^A and B^+*e*^B, whereas the right configuration is the parallel combination of A^+*e*^A and B^+*e*^B.

As stated above, there are only four different s-contacts. They are depicted in Figure 8A in a less schematic representation than we used before. A chain entanglement is considered as a connection of s-contacts. Circuit topology allows only three kinds of connections: in series, in parallel, in cross. In-series and in-parallel connections can also be concerted. In the present paper we lay down the foundation of this new approach to entanglement, and therefore we consider only fairly simple cases. To avoid the impression of a too simplistic consideration, Figure 8B shows that just two contacts in cross already make quite a messy chain. We expect that it is not immediately clear why these chains are described by the strings provided, although the definition provided in section “S-Contacts” is sufficient. Such questions of practical application of our approach to various systems will be considered in our next papers.

### Quantification of a String

In order to describe a chain completely, one has to know coordinates of all its parts and which parts are connected to each other. However, this complete information is hard to analyze to make useful conclusions. It is a common idea in statistical physics to lose some extra information obscuring the essence of the phenomenon. When we introduced a string based on the chain coordinate file, we lost some information, such as the spatial distance between contacts, but gained the ability to analyze it by the circuit topology formalism. In this case, the loss of information can be cushioned by naming contacts not with letters but with the contact site coordinates along the chain, e.g., AA→(38,214)(38,214). In this section, we will continue to lose information. This notation is harder for a human to follow, but easy for a computer, especially when there are more contacts in a string than letters in the alphabet. Thus, a computer can have two strings of data: one for hard contacts and one for soft contacts. Depending on the task, they can be analyzed independently, for instance, if we are interested in chain entanglement only. Alternatively, both strings of data can be combined to see the interplay of h- and s-contacts, which should be straightforward for a computer (not for a human) because the coordinates of contact sites are already an internal part of the strings.

Let us temporarily forget about string subscripts and consider only string lines. Also, we will not distinguish between h- and s-contacts. In principle, the difference is important, but not for the purpose of this section. We define a circuit as a segment of a string that consists only of pairs of letters. For example, AA, ABAB, ABCCAB are circuits, whereas ABCABD is not a circuit. Larger circuits resemble separate messy blobs that are colloquially called knots. We will not use this term in order to avoid confusion and interference with mathematical theory of knots. The number of pairs in a circuit is called the circuit order. A σ-algebra on a string is a collection of all possible circuits on this string, including an empty set, i.e., the absence of circuits, and the string itself as a circuit. To define the string size, we write down the number of its circuits of each order from right to left. The right-most digit is the number of one-pair-circuits, the next digit is the number of two-pair-circuits, and so on. Basically, we just list the structural units of the string. In case of two contacts, ABAB is size [10], ABBA is [11], AABB is [12]. The number of digits matches the number of contacts, which is the circuit-order of the whole string. The left-most digit is always 1.

Let us consider a chain with *n* contacts. Its minimal size is [100⋯0], which corresponds to all contacts in cross, e.g., ABCDE ABCDE. Its maximal size is [123⋯n], which means that all contacts are in series, e.g., AA BB CC DD EE. If all contacts are in parallel, the size is [111⋯11]. Not everything in this range is possible, for instance, [120] is impossible. Singling out the impossible configurations can be done by combinatorics and is beyond the scope of this paper. Different strings can have the same size; therefore, by introducing the string size we lose some, but not all, information about the specific arrangement of contacts, or structural units, of the chain. In other words, one cannot deduce the string from its size only.

Each string can be represented by a point in an infinite dimension coordinate space where each axis corresponds to a circuit order. For example, the coordinate along the axis corresponding to circuit order three gives the number of circuits consisting of three pairs of letters. Although the coordinate space is formally infinite dimension, it is clear that the maximal circuit order is the number of contacts in the string. The coordinates on the axes corresponding to higher circuit orders are 0. We make this space a metric space by introducing the distance, *d*, between strings, according to

(Equation 1) $$d\left(X,Y\right)\equiv \sqrt{\sum_{k=1}^{\infty }k^{2}{\left(x_{k}-y_{k}\right)}^{2}}\text{,}$$

where *k* is the circuit order, *x* and *y* are the corresponding coordinates of the strings. For example, let us find the distance between AABBCC and ABCCAB. The first string has three circuits order 1 (i.e., consisting of one letter, k=1), namely, AA, BB, and CC; two circuits order 2: AABB and BBCC; and one circuit order 3, which is the string itself AABBCC. Hence, its size is [123]. The other string has one circuit order 1, CC, and one circuit order 3, which is the sting itself, ABCCAB. Hence, its size is [101]. The distance between the strings $d\left(123,101\right)=\sqrt{3^{2}{\left(1-1\right)}^{2}+2^{2}{\left(2-0\right)}^{2}+1^{2}{\left(3-1\right)}^{2}}=\sqrt{20}$. For comparison, Euclidian space has all axes order 1. The circuit order *k* in Equation 1 gives higher weight to higher order circuits. The distance along an axis is proportional to this axis order, which explains the choice of the power of *k* in Equation 1. For example, $d\left(600,400\right)=\sqrt{3^{2}{\left(6-4\right)}^{2}}=3\cdot \left(6-4\right)$ is proportional to 3.

A mathematical distance must satisfy the following: (1) d(X,X)=0; (2) d(X,Y)>0 if X≠Y; (3) d(X,Y)=d(Y,X); (4) d(X,Z)≤d(X,Y)+d(Y,Z). The first three are obvious from Equation 1. To prove the last one, we can consider the squares, because distance is non-negative.

(Equation 2) $$d^{2}\left(X,Z\right)=\sum_{k=1}^{\infty }k^{2}{\left(\left(x_{k}-y_{k}\right)+\left(y_{k}-z_{k}\right)\right)}^{2}=d^{2}\left(X,Y\right)+d^{2}\left(Y,Z\right)+2\sum_{k=1}^{\infty }k^{2}\left(x_{k}-y_{k}\right)\left(y_{k}-z_{k}\right)\text{.}$$

(Equation 3) $${\left(d\left(X,Y\right)+d\left(Y,Z\right)\right)}^{2}=d^{2}\left(X,Y\right)+d^{2}\left(Y,Z\right)+2d\left(X,Y\right)d\left(Y,Z\right)\text{.}$$

Hence, we should show that

(Equation 4) $$\sum_{k=1}^{\infty }k^{2}\left(x_{k}-y_{k}\right)\left(y_{k}-z_{k}\right)\leq \sqrt{\sum_{k=1}^{\infty }k^{2}{\left(x_{k}-y_{x}\right)}^{2}\sum_{l=1}^{\infty }l^{2}{\left(y_{l}-z_{l}\right)}^{2}}\text{.}$$

Let us introduce $a_{k}\equiv k\left(x_{k}-y_{k}\right)$ and $b_{k}\equiv k\left(y_{k}-z_{k}\right)$. Equation 4 becomes

(Equation 5) $$\sum_{k=1}^{\infty }a_{k}b_{k}\leq \sqrt{\sum_{k=1}^{\infty }a_{k}^{2}\sum_{l=1}^{\infty }b_{l}^{2}}\text{,}$$

which holds owing to the Cauchy-Bunyakovsky-Schwarz inequality (note, the CBS inequality has the absolute value on the left hand side; therefore, it is stronger than Equation 5). Hence, our triangle inequality holds as well and *d* is indeed a metric.

We define the string roughness as the distance from the string to an “empty” string, which describes a disentangled chain,

(Equation 6) r(X)≡d(X,0).

Note that in general d(X,Y)≠d(X,0)−d(Y,0)=r(X)−r(Y), which is also the case in Euclidian space due to the triangle inequality. A string roughness contains less information than a string size does, but it helps compare entanglement or messiness of different chains. The roughness of a disentangled chain is 0. *r*(ABAB) $=\sqrt{4}$, *r*(ABBA) $=\sqrt{5}$, *r*(AABB) $=\sqrt{8}$. A blob ABAB is less rough than a sequence of two h-loops AABB. In case of two contacts the difference is not that large. However, a blob of, say, 7 contacts looks similar to a blob of 5 or 6 contacts. But two blobs of 5 and 2 contacts in series look quite different from one blob of 7 contacts. For a chain with *n* contacts,

**Table:** 

*n* contact are	String Size	String Roughness
All in cross	[100⋯00]	*n*
All in parallel	[111⋯11]	${\left(\frac{n\left(n+1\right)\left(2n+1\right)}{6}\right)}^{\frac{1}{2}}$
All in series	[123⋯n]	${\left(\frac{n\left(n+1\right)\left(n^{3}+4n^{2}+6n+4\right)}{30}\right)}^{\frac{1}{2}}$

Since the ranges for different *n* overlap, chains with a smaller number of contacts might still be rougher than chains with a larger number of contacts. Three contacts give rise to 15 different strings, 3 pairs out of which are symmetric with respect to orientation, namely, AABCBC and ABABCC, AABCCB and ABBACC, ABACCB and ABBCAC. Indeed, each pair reads in the topological sense the same from left to right and from right to left. This leaves 12 independent configurations:

$$\begin{array}{ccc} \text{AABBCC}\left[123\right]\left(\sqrt{34}\right) & \text{ABCCBA}\left[111\right]\left(\sqrt{14}\right) & \text{ABACBC}\left[100\right]\left(\sqrt{9}\right)\\ \text{ABBCCA}\left[122\right]\left(\sqrt{29}\right) & \text{ABCBCA}\left[110\right]\left(\sqrt{13}\right) & \text{ABCBAC}\left[100\right]\left(\sqrt{9}\right)\\ \text{AABCCB}\left[112\right]\left(\sqrt{17}\right) & \text{ABBCAC}\left[101\right]\left(\sqrt{10}\right) & \text{ABCACB}\left[100\right]\left(\sqrt{9}\right)\\ \text{AABCBC}\left[111\right]\left(\sqrt{14}\right) & \text{AABBCC}\left[101\right]\left(\sqrt{10}\right) & \text{ABCABC}\left[100\right]\left(\sqrt{9}\right) \end{array}$$

One can see that smaller blobs are less rough than larger blobs. And several blobs (or loops) are more rough than fewer similar blobs (or similar loops). So far, these results coincide with intuitive expectations. However, a further investigation of the interplay between blob sizes and blob numbers, which would keep the roughness constant is needed.

So far, we have considered only a string line. Now, we account for string subscripts. The definitions and everything else are absolutely the same. So, AA is a circuit, but A_*B*_A is not a circuit. The addition of subscripts reduces the number of circuit but cannot change the circuit order of the whole string, i.e., the number of digits in the string size. A comparison of string sizes with and without subscripts gives the contribution of h-h and h-s contact interaction.

## Discussion

In this paper, we took another step in the development of circuit topology. Our approach is self-consistent and independent from other approaches. However, it is always a good idea to put it in the context of different theories.

### Comparison with Knot Theory

A common approach to description of a chain topology is based on mathematical knot theory. Here, we do not aim at a detailed comparison but will show how to describe our strings in terms of knot theory and will calculate an important knot invariant called the Alexander polynomial. The main ideological difference between the approaches is that circuit topology works with chains in 3D and does not take any 2D projections (the flat diagrams in Figures 3D–3F are shown for visualization; they are not a part of the theory).

Knot theory works with mathematical knots that are closed structures and can be made by joining the ends of a chain. Mathematical knots are considered by means of their 2D projections, the so-called knot diagrams. Two knot diagrams belong to the same mathematical knot if and only if they can be turned into each other by a series of Reidemeister moves. Because knot theory can only describe chain entanglement, here we will not consider h-contacts and subscripts that arise due to h-contacts. Subscripts can appear with s-contacts in cases like slip-knots, which can be eliminated by Reidemeister moves. In other words, slip-knots are not accounted in knot theory but accounted in circuit topology. Subscripts can also appear at chain ends similar to the right tail in Figure 6C. But such subscripts will turn into s-contacts when the ends of a chain are joined together to form a mathematical knot. Therefore, here we will consider only s-contacts.

There are only four possible s-contacts, A^+*e*^A, A^+*o*^A, A^–*e*^A, A^–*o*^A, whose combinations are described by circuit topology. The first two are shown in Figure 3; the last two are their asymmetric counterparts where all the crossings are flipped. Knot theory considers so-called nugatory crossings, which are crossings in a knot diagram that can be flipped without changing the knot (Torisu, 1999). In the language of circuit topology, a flip of such a crossing corresponds to a change of chirality of the s-contact.

To turn s-contacts into mathematical knots, one has to join their ends. The resulting knot matrices and Alexander polynomials are

$$A^{+e}A\to \left(\begin{array}{cccc} -1 & t & 1-t & 0\\ 1-t & -1 & t & 0\\ t & 0 & 1-t & -1\\ 1-t & 0 & -1 & t \end{array}\right)\;\text{Δ}\left(t\right)=t^{2}-t+1$$

$$A^{-e}A\to \left(\begin{array}{cccc} 1-t & t & -1 & 0\\ 1-t & 0 & t & -1\\ t & -1\; & 1-t\; & 0\;\\ -1 & 0 & 1-t & t \end{array}\right)\;\text{Δ}\left(t\right)=t^{2}-t+1$$

$$A^{+o}A\to \left(\begin{array}{cccc} -1 & t\; & 1-t\; & 0\;\\ -1 & 1-t & 0 & t\\ 0 & t & -1 & 1-t\\ 1-t & 0 & -1 & t \end{array}\right)\;\text{Δ}\left(t\right)=t^{2}-3t+1$$

$$A^{-o}A\to \left(\begin{array}{cccc} 1-t & t\; & -1\; & 0\;\\ -1 & t & 0 & 1-t\\ 0 & 1-t\; & -1\; & t\;\\ -1 & 0 & 1-t & t \end{array}\right)\;\text{Δ}\left(t\right)=t^{2}-3t+1$$

In these matrices, rows correspond to crossings and columns correspond to arcs. The crossings and arcs are numbered from left to right along the chain. The arc number 1 is the joining of the chain ends. Arcs begin and end when they go below crossings. Matrix elements for right-handed crossings are –1 if the arc ends at the crossing, *t* if the arc begins are the crossing, and 1–*t* if the arc goes over the crossing, otherwise zero. All crossings in A^+*e*^*A* are right handed. Matrix elements for left-handed crossings are *t* if the arc ends at the crossing, –1 if the arc begins are the crossing, and 1–*t* if the arc goes over the crossing, otherwise zero. All crossings in A^–*o*^*A* are left handed. Deleting one arbitrary row and one column and counting the determinant of the resulting matrix gives the Alexander polynomial, which is defined up to $\pm t^{k}$, where *k* is an arbitrary integer. The mathematical knot corresponding to A^+*e*^*A* and A^–*e*^*A* is the trefoil; the mathematical knot corresponding to A^+*o*^*A* and A^–*o*^*A* is the figure eight knot.

**Figure 9:**
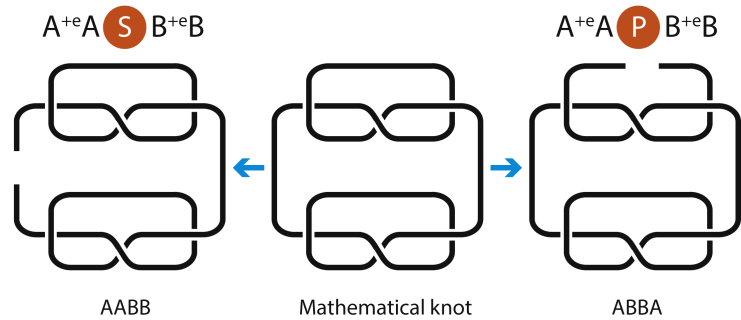
The Ambiguity of a Chain Representation by Knot Theory The mathematical knot in the middle panel can be cut on a side, forming the chain in the left panel, or it can be cut at the top, forming the chain on the right panel. The two chains are visually different and give rise to different strings.

If a string can be split into circuits, one has to only find the Alexander polynomials of each circuit and multiply them because the polynomial of a sum of knots is a product of polynomials of each knot. Any circuit consisting of s-contacts in series and/or in parallel can be viewed as a sum of knots because they do not create new crossings and one can always single out a knot at the diagram of the joined knot. Therefore, the Alexander polynomial of such a circuit is a product of the polynomials of s-contacts in the circuit. In other words, if no contacts are in cross, there is no need to derive the knot matrix. It also means that *n* even s-contacts and *m* odd s-contacts produce the same knot no matter how the contacts are arranged, i.e., all such knots can be deformed to each other by a sequence of Reidemeister moves. The circuit topology obviously distinguishes all such configurations. This statement can be intuitively visualized by tying two s-contacts in series, joining the ends and cutting one of the s-loops, which gives two s-contacts in parallel, see Figure 9.

Segments of a string with s-contacts in cross do not allow such a treatment and have to be dealt with differently, for example, ABBCDDAC, where contacts A and B are in parallel, contacts B and C are in series, but the treatment from the previous paragraph is impossible due to contacts A and C which are in cross. Figures 3B and 3E, although equivalent, have a different number of crossings but lead to the same Alexander polynomial. We choose Figure 3E because we want to use them as blocks 4X4 in order to describe contact relationships. Two contacts give rise to 2⋅4=8 crossings, which generate an 8X8 knot matrix. The idea is to fill the new matrix of the joined knot with the blocks of original contacts with some minor rearrangements depending on the contact types. Thereby, one could move along a string and add new rows and columns to the knot matrix, according to solid rules established for each contact type. Here we will not derive all such rules but will show how to do it.

Let us start with two s-contacts in series. There could be A^+*e*^AB^+*e*^*B*, A^+*e*^AB^–*e*^*B*, A^–*e*^AB^–*e*^*B*, A^+*e*^AB^+*o*^*B*, A^+*e*^AB^–*o*^*B*, A^–*e*^AB^+*o*^*B*, A^–*e*^AB^–*o*^*B*, A^+*o*^AB^+*o*^*B*, A^+*o*^AB^–*o*^*B*, A^–*o*^AB^–*o*^*B*. The difference between them is how arcs join together s-contacts. Let us consider A^+*e*^AB^+*o*^*B*:

$$A^{+e}AB^{+o}B\to \left(\begin{array}{cccccccc} | & | & | & | & & & & \\ \left(\right) & | & | & | & \left[\right] & & & \\ \left(\right) & | & | & | & \left[\right] & & & \\ \left(\right) & | & | & | & \left[\right] & & & \\ & & & & | & | & | & |\\ \left[\right] & & & & \left(\right) & | & | & |\\ \left[\right] & & & & \left(\right) & | & | & |\\ \left[\right] & & & & \left(\right) & | & | & | \end{array}\right)$$

The rows 1 to 4 correspond to the rows (i.e., crossings) of the first contact; rows 5 to 8 are from the second contact. The same stays for the columns (i.e., arcs). Hence, the matrix of A^+*e*^A is in the top left corner and the matrix of A^+*o*^A is at the bottom right corner. We do not write the values of matrix elements, but rather which elements are unchanged and how other elements change. The arcs number 2, 3, 4 of A^+*e*^A are not affected by the joining and are shown with |. Likewise, the arcs number 2,3,4 of A^+*o*^A are not affected and become arcs number 6,7,8. Each resulting knot consists of two parts: the joining of the s-contacts and the joining of the ends of the s-contacts. The first part transfers the three elements of the first column marked by round brackets () to the column 5 marked by square brackets []. The second part transfers the three elements of the column 5 marked by () to the column 1 marked by []. All the other elements of the matrix are 0.

Let us now consider two s-contacts in parallel, A^–*o*^B^–*o*^BA. To join them, we cut the arc at the bottom of contact A and join the cutoffs with the ends of contact B, compare with Figure 4.

$$A^{-o}B^{-o}BA\to \left(\begin{array}{cccccccc} | & | & | & & & & & |\\ | & | & \left(\right) & & & & \left[\right] & |\\ | & | & \left(\right) & & & & \left[\right] & |\\ | & | & \left(\right) & & & & \left[\right] & |\\ & & | & | & | & | & & \\ & & | & | & | & | & & \\ & & | & | & | & | & & \\ & & \left(\right) & | & | & \left[\right] & & \end{array}\right)$$

Note that the knot joining opening, i.e., the AB part of the ABBA, is in the top half of the matrix, whereas the knot joining closure, i.e., the BA part of the ABBA, is in the bottom half of the matrix. If the contact joining closure occurs later, e.g., as in ABBCAC, then the corresponding shift of the matrix element also occurs after the shifts for contact C have been applied.

**Figure 10:**
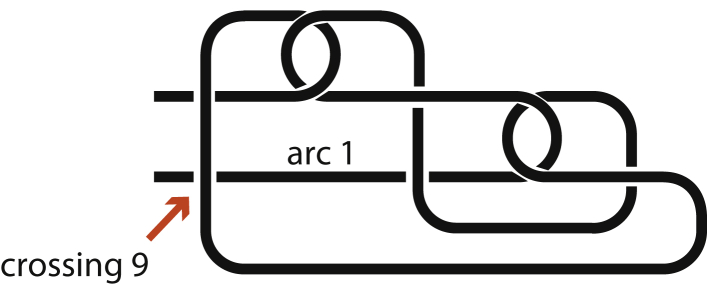
Soft Contacts Connected in Cross Give Rise to an Extra Crossing Two s-contacts in Cross, A^+*e*^B^+*o*^AB, where contact A is at the right bottom corner. The s-contacts cannot be separated. To form a knot, one has to join into an arc the chain ends pointing to the left. Crossing 9 is a new crossing, which is needed to join together the chain ends.

Two s-contacts in cross are different from s-contacts in series and in parallel because one cannot join the ends without creating a new crossing that changes its Alexander polynomial. This is the reason why Alexander polynomials of s-contacts in cross do not multiply. As depicted in Figure 10, two s-contacts in cross give rise to 9 crossings, instead of 8 crossing for in-series and in-parallel configurations. That new extra crossing, called crossing 9, is needed to join the chain ends together. Which chain segment goes above or below in this crossing is ambiguous and depends on our choice. We chose it as in Figure 10 because otherwise a new s-contact would be created because the left-most vertical segment would be enveloped. This ambiguity is a specific case of a common problem of turning a chain into a mathematical knot.

The new crossing and the corresponding new arc are described by the last row and column in the resulting matrix:

$$A^{+e}B^{-o}AB\to \left(\begin{array}{cccccccc} | & | & \left(\right) & & \left[\right] & & & \\ \left(\right) & | & | & & & | & \left[\right] & \\ \left(\right) & | & | & & & | & \left[\right] & \\ \left(\right) & | & \left(\right) & & \left[\right] & | & \left[\right] & \\ & & | & | & & \left[\right] & | & \\ & & | & | & & | & | & \\ & & | & | & & | & | & \\ & & \left(\right) & | & & | & | & \end{array}\right)$$

The rules for other combinations of s-contacts can be found in a similar manner. Therefore, any string segment can by automatically translated into a knot matrix. However, if a string contains lots of contacts in cross, the resulting matrix rank can be too large to handle comfortably. The complexity of the string notation does not grow with the number of contacts.

### Extended Contact Order

Another commonly used measure for a chain comes from protein biophysics. An important characteristic of proteins is the contact order, which is defined as the average sequence distance between residues that form contacts in a folded protein, normalized by the protein length. Contact order is defined for h-contacts only. Naming contacts with letters is pleasant for the eye but leads to a loss of the information essential for evaluation of the contact order. However, if contacts are named as the corresponding coordinates along the chain, e.g., protein residue numbers, then the contact order is calculated directly from the string. Importantly, the presented circuit topology framework allows us to define contact order for s-contacts as well. The corresponding calculation is mathematically equivalent to that for h-contacts. Unlike h-contact sites, s-contact sites are not fixed and can slide freely along the chain. Therefore, a change of the s-contact order can be a measure of a chain deformation. A lower s-contact order means tighter, closer packed blobs with higher chain curvature. A higher s-contact order means a relax winding of the chain with lots of unoccupied space between segments. A relax winding means less restrictions on motion of different segment, which in turn imply higher entropy. Hence, the s-contact order might be related to the chain entropy.

A sequence of s-contacts in cross form a blob-like structure that is sensitive to bulk deformation, whereas s-contacts in parallel form a rod-like structure that can band but is unlikely to change its volume. These are examples of higher-order structures above simple contact-contact relations. The circuits introduced in the previous chapter is also a higher-order structure. Such structures are seen as patterns on circuit topology matrices (Scalvini et al., 2020). Circuit topology is applicable to such structures as well. Chains consisting of easy-to-deform structures can be easier contracted into a confined space or squeezed through a narrow channel. For comparison, studies performed for h-contacts (Heidari et al., 2017) (Satarifard et al., 2017) showed that external confinement favors certain topological structures. Diffusion of polymers through membrane channels or polymer external entanglement are associated with entropy terms, which is another sign that s-contact order and entropy are related.

### Other Measures

Homogeneity of contact positions can be readily quantified using the circuit topology approach. n contacts give rise to 2n contact sites. If they are equally distanced from each other and from the chain ends, the distance between them is L/(2n+1), where *L* is the chain length.${\left(\frac{L}{2n+1}\right)}^{2n+1}$ is the maximum value of the product of distances between contact sites (and chain ends) for an arbitrary distribution of contact sites. Calculation of this product normalized by its maximal value gives a measure of the homogeneity of contact positions, ranging from 0 to 1, where smaller values correspond to clustering of contacts. This can be calculated for h- and s-contacts independently. Clustering implies that a chain will not deform equally in different parts if pushed or pulled.

One can also consider an interplay of h- and s-contacts, which might be relevant for logical chains, when some system can be formally mapped to a linear chain of sorted objects. H-contacts would be not due to a chemical bond but because of some logical proximity of chain units, whose definition depends on the system considered. A proximity is described by s-contacts. In case of clustering, clusters of higher s-contact order have higher chances of formation of h-contacts. The shortest distance between chain units is reduced owing to h-contacts, which can be viewed as shortcuts and readily accessible in the string notation.

Circuit topology offers new measures to compare different chains based on the type and amount of the chain structural units. We introduced a chain size as a list of structural units and a mathematical distance between chains as a measure of difference in structural units of the chains. A chain roughness gives a quantitative measure of how far a chain is from a disentangled chain. At the first sight, these measures adequately describe a chain entanglement. Now, the new slab of the theory should be tested against real molecular chains and mathematical logical chains.

Since we are particularly interested in biomolecules, we are planning a follow-up paper with an extensive study of the interplay between biological functions of real molecules and their topological properties analyzed in terms of circuit topology. Here, we give a simple example that can be traced with a naked eye, the convenience unavailable for large molecules with hundreds of contacts. H-contacts of YibK methyltransferase were found in Scalvini et al. (2020) in the form of a topology matrix, which can be straightforwardly translated to a string. From its 3D structure one can see that it also has 1 s-contact. The string reads (**AB**)C(**BDE**)(**EF**)(**FDG**)S^+*e*^(**HI**)(**IGA**)**H**S**C**. The parentheses denote concerted contacts; see section “Results”. This type of s-contact, Figure 3B, corresponds to a trefoil knot, which YibK is known to consist of. Ordering concerted contacts, one has to avoid cross configurations. Hence, the strings size is [1000000113]; the roughness is $\sqrt{122}$. There are no subscripts in the string. The string has one circuit, **DEEFFD**.

### Limitations of the Study

We have presented a development of the theory of circuit topology. In particular, we suggested a conceptually new approach to description of linear chain entanglement. With these extensions, circuit topology refers to the arrangement of contacts and contact-contact interactions within a folded chain, where contacts represent constraints of various origins (i.e., s-contacts and h-contacts). One of the outcomes is a possibility to translate both contacts and knots into the language of combinatorics. We also touched an issue of disentangling a chain, such as a treatment of slip-knots. Our approach to topological description is not only useful for structural classification but also gives quantitative measures that can be tested for their correlation with system's properties. In the past, we showed how h-contact order and frequencies of P, X, S for h-contacts correlate with folding rate of a polymer and how the circuit topology relation frequencies for h-contacts can be used to interpret experimentally measured single molecule mechanical unfolding data. One can readily extend those studies to s-contacts and h-h interactions. Furthermore, the circuit topology approach might allow for engineering molecules using topological units and circuit topology operations.

In our model, we consider only topological properties of a chain. However, the 3D conformation of a real chain can be affected by its physical properties, which could limit topological possibilities. One example is a persistence length of the chain, which is especially important for processes when chain segments have to pass through loops, e.g., entangling/disentangling. Our model chain is smooth and infinitely thin. Real chains have excluded volume and side chains, which in turn pose geometrical constraints.

In this study, we focused on isolated chains; however, extensions to multi-chains and supramolecular structures are possible. Indeed, circuit topology is a multi-level approach. The lowest level of structuring is the three basic structures: in series, in parallel, in cross. The next level is the level of circuits introduced in section “Quantification of a String”. Circuits have different levels of detalization as well, depending on their size of roughness. The topology of circuits is a barcode of a molecule, hence the name “circuit topology.” A higher level of structuring is the level of single molecules hooked to each other. Such connections give rise to s-contacts between molecules, which can be treated in a similar manner as s-contacts between circuits. This is a matter for further investigation.

In this article, we consider a static chain only; however, an extension to some dynamic problems is possible. In fact, circuit topology has been previously used to study molecular folding processes (Heidari et al., 2020). Those studies can be readily extended to include s-contacts. Although topological factors are critically important in folding, they are not the only determinants of folding. Contact energy, loop sizes, and steric constraints are not included in this article, but they also determine folding pathways and folding kinetics. Cooperative folding and unfolding can also be seen in experiments and are partly captured by the circuit topology framework. For example, parallel contacts facilitate formation of each other owing to zipping effects. Concerted contacts may show significant cooperativity. However, a full analysis of folding processes requires an approach that combines topology with other physical determinants of folding. These topics will be discussed in future studies.

In section “S-Contacts”, we mentioned a slip-knot A^+*o*^A_+*A*_ and speculated what an h-contact on the slip-loop, i.e., A^+*o*^A**BB**_+*A*_, would prevent it from disentangling by making it harder for the chain to pass through the loop. One can make one step further and consider a whole chain sliding among other chains. This process is called reptation. The dynamics of the process, at the current state of theory development, cannot be described by circuit topology (neither by knot theory) because it creates new s-contacts and new subscripts. However, it might be related to some extent via chain size and roughness and via s-contact order, letting to expect which chains might be more prone to reptation.

In case of logical chains, a possible limitation is due to the presence of non-pair-wise contacts, i.e., concerted contacts. We do account for them to some extent by considering them as a set of pairwise contact. However, if a concerted contact has to be split into too many pairwise contacts, such a description might not be adequate. A non-fundamental semi-limitation is the need for computational processing and proper algorithm to extract h- and s-contacts and to analyze the string. In case of small molecules, it can be done and verified by a naked eye, but a bit larger molecules already require computer assistance.

### Resource Availability

#### Lead Contact

Alireza Mashaghi, Medical Systems Biophysics and Bioengineering, Leiden Academic Centre for Drug Research, Faculty of Science, Leiden University, 2333CC Leiden, The Netherlands; Email: a.mashaghi.tabari@lacdr.leidenuniv.nl.

#### Materials Availability

This study did not generate new unique reagents.

#### Data and Code Availability

The published article includes all datasets generated or analyzed during this study.
